# High-throughput screening system for dynamic monitoring of exocytotic vesicle trafficking in mast cells

**DOI:** 10.1371/journal.pone.0198785

**Published:** 2018-06-08

**Authors:** Takeshi Kiyoi, Shuang Liu, Muhammad Novrizal Abdi Sahid, Masachika Shudou, Kazutaka Maeyama, Masaki Mogi

**Affiliations:** 1 Department of Pharmacology, Ehime University Graduate School of Medicine, Shitsukawa, Toon, Ehime, Japan; 2 Division of Analytical Bio-medicine, Advanced Research Support Center, Ehime University, Shitsukawa, Toon, Ehime, Japan; 3 Department of Pharmaceutical Chemistry, Faculty of Pharmacy, Gadjah Mada University, Sekip Utara, Yogyakarta, Indonesia; Cornell University, UNITED STATES

## Abstract

Mast cells, in addition to endocrine cells and neurons, are typical secretory cells. Their function in allergic inflammation is to secrete inflammatory mediators from secretory vesicles. Intracellular synthesized inflammatory mediators are transported by vesicular monoamine transporters (VMATs) to vesicles where they are stored. After stimulation, the contents of the secretory vesicles are released via exocytosis. This study established a high throughput imaging screening system to monitor the functions of secretory vesicles in mast cells, including molecular uptake via VMAT2 and the exocytotic process, by using a novel fluorescent probe, FFN206, which was developed as a VMAT2 substrate. After loading with FFN206, the rapid uptake of FFN206 was observed and secretory vesicles in mouse bone marrow derived mast cells and a cultured mast cell line were clearly visualized. FFN206 uptake by secretory vesicles was time-dependent and was blocked by reserpine. Furthermore, exocytotic trafficking was monitored dynamically by real-time high-throughput fluorescence quantitation. In the present study, we verified the application of FFN206 for the monitoring of functional vesicles. This high-throughput screening system may benefit instinctive drug evaluation.

## Introduction

Mast cells, in addition to exocrine cells, endocrine cells and neurons, are typical secretory cells. They are involved in the innate and adaptive immune systems and their roles in allergic and anaphylactic reactions are well characterized [1, 2]. Vesicles in mast cells take up and store mediators such as biological amines, peptidoglycan, chymase and tryptase [3]. The granule content is important in determining the fate of granule formation and maturation [4, 5]. The characteristics of vesicle contents and cell activity in mast cells have been widely studied to clarify the mechanisms underlying their pathological role.

Mast cells release their vesicle contents by an exocytotic process that is activated via a high affinity immunoglobulin E receptor (FcεRI)-mediated signal pathway, Ca^2+^ ionophore or other peptides [6]. Alterations in the amount, location and kinetics of the released vesicles have profound consequences on the physiological function of mast cells. Vesicles accumulate beneath the plasma membrane and undergo membrane fusion, which is orchestrated by the interaction of soluble N-ethylmaleimide-sensitive factor attachment protein receptor (SNARE) proteins [7]. Monitoring of the dynamic capabilities of exocytosis is critical for the assessment of mast cell function.

The screening of mast cell exocytotic function is often conducted using biochemical approaches. For example, the measurement of released mediators such as histamine is an effective method to dissect mast cell function. Histamine is produced by histidine decarboxylase and taken up from the extracellular space through cation transporters on the plasma membrane of mast cells [8–10]. Histamine in the cytoplasm is transported into vesicles via vesicular monoamine transporter 2 (VMAT2) [11] and the histamine content of the granules is maintained by the expression of VMAT2 [3, 12]. In addition, high-performance liquid chromatography (HPLC) is used for the accurate analysis of histamine release at individual time points. However, it is not a dynamic monitoring method. Therefore, dynamic monitoring techniques such as fluorescently labeled dextran or β-hexosaminidase fused to pHluorin (β-Hex-pHl) have been developed for exocytotic observation [13, 14].

Recently, fluorescence false neurotransmitters (FFNs) were developed to evaluate the transport function of VMAT2. For example, FFN511 and FFN102 were used to visualize dopaminergic synapses and activity in brain slices [15]. However, they are not suitable for mast cells because FFNs are not taken up into cells in culture or are pH sensitive [16]. FFN206 was designed as a fluorescent probe for VMAT2 based on the combination of an aryl ethylamine fragment with a photostable fluorescent system (7-amino-coumarin), which is not pH sensitive [17]. FFN206 is transported into the cytoplasm from the extracellular space via the dopamine transporter organic cation transporter 3 (OCT3) and plasma membrane transporter. This compound is frequently used in the monoamine research of neurons [18].

In the present study, we tried to visualize mast cell vesicles through labeling of FFN206, considering that the expression of both OCT3 and VMAT2 is also observed in mast cells. Furthermore, a high-throughput system for the dynamic screening of the amine transport system (uptake and release) function and exocytotic trafficking by the quantitative analysis of real-time fluorescence images was established. By using this system, large-scale imaging-based dynamic screening for exocytotic function in mast cells can be realized.

## Materials and methods

### Animals

C57BL/6^+/+^ and C57BL/6^*bg/bg*^ mice were purchased from Japan SLC, Inc. (Hamamatsu, Japan), and each strain was maintained by mating between the same strains. Heterozygous C57BL/6^*bg/+*^ mice were obtained by mating male *bg/bg* mice with female +/+ mice. Male mice aged between 10 to 16 weeks were used for all experiments. The animals were housed at a constant temperature of 22 ± 2°C, humidity of 55 ± 10% and an automatically controlled 12:12 h light-dark cycle with lights on at 7:00 A.M. Food and water were provided *ad libitum*. The experimental protocols were performed in accordance with the guidelines of the Animal Care Committee of Ehime University and were approved by the University Committee for Animal Research.

### Preparation of bone marrow-derived mast cells (BMMC)

BMMC were collected from the femur and tibia of mice and differentiated as reported previously [19]. In brief, BMMC were cultured in RPMI1640 containing 15% fetal calf serum, 50 U/ml penicillin, 50 μg/ml streptomycin and 5 ng/ml IL-3 for 4 weeks. BMMC maturation was verified by staining with safranin and the expression of the FcεRI receptor and c-kit receptor was confirmed by flow cytometry. The recovered cells were washed with PIPES buffer and attached to glass slides by cytospin. The specimens were stained with Alcian blue and safranin after fixing in Carnoy’s solution for 30 min and observed under a light microscope (BZ-9000, KEYENCE, Tokyo, Japan). Fc receptors were blocked for flow cytometric analysis by incubating cells with an anti-mouse CD32 antibody (Thermo Fisher, Tokyo, Japan) at 1.0 μg per 10^6^ cells in 100 μl for 5–10 min on ice, prior to immunostaining. After washing, the cells were incubated with FITC-labeled anti-mouse FcεRI receptor antibody (1:100), PE-labeled anti-mouse c-kit receptor antibody (1:100) or their respective isotype-matched control antibodies (all from Thermo Fisher, Tokyo, Japan) for 1 h at room temperature. The cells (10,000 cells per sample) were collected and analyzed with a BD FACSCalibur flow cytometer and CellQuest software (BD Biosciences, Tokyo, Japan). Both FcεRI- and c-kit-positive cells were considered functional mast cells.

### FFN206 loading of RBL-2H3 cells and BMMC

Rat basophilic leukemia cells (RBL-2H3) (10^5^ cells/ml) were cultured in Minimum Essential Medium (MEM) containing 15% fetal calf serum and antibiotics (penicillin-streptomycin) in a 96-well plate (Corning, Tokyo, Japan). To label the secretory vesicles in BMMC and RBL-2H3 cells, a final concentration of 5 μM FFN206 (Abcam, Tokyo, Japan) in PIPES buffer containing 119 mM NaCl, 5 mM KCl, 25 mM PIPES, 5.6 mM glucose, 0.4 mM MgCl_2_, 1 mM CaCl_2_, 40 mM NaOH and 0.1% BSA (pH 7.2) was used as the loading buffer. After loading at 37°C for 90 min, FFN206-labeled cells were washed using PIPES buffer and used for further detection or subjected to further analysis without washing. Confocal observation microscopy was performed at room temperature with a Nikon A1 microscope (Nikon, Tokyo, Japan) equipped with a laser (excitation: 405 nm, emission: 425–475 nm). The images were processed with NIS-Elements Ar (Nikon, Tokyo, Japan).

For co-labeling using FFN206 and Alexa568-dextran, an established secretory vesicle indicator [13], RBL-2H3 cells (10^5^ cells/ml) were pre-cultured in complete MEM containing 1 mg/ml of Alexa fluor 568-dextran (Thermo Fisher, Tokyo, Japan) in a glass bottom dish for 24 h at 37°C. Then, the culture medium was replaced with fresh complete MEM containing 5 μM FFN206 and the cells were incubated for 90 min at 37°C. After loading, cells were washed two times with PIPES buffer and the localizations of FFN206 and Alexa568-dextran were observed using a Nikon A1 microscope (Nikon, Tokyo, Japan) equipped with a laser (excitation: 562 nm, emission: 570–620 nm; excitation: 405 nm, emission: 425–475 nm, respectively). The images were processed with NIS-Elements Ar (Nikon, Tokyo, Japan).

### Visualization of secretory vesicles in RBL-2H3 cells

RBL-2H3 cells were seeded on a 96-well culture plate one day before the imaging experiment. Thapsigargin (TG), a sarcoendoplasmic reticulum Ca^2+^-ATPase pump inhibitor, was added at the indicated doses to stimulate mast cells through the intracellular Ca^2+^-dependent signaling pathway. Reserpine, a VMAT2 inhibitor, was added to the cells at the indicated doses (from 10^−1^ to 10^5^ nM) for 2 h before FFN206 loading.

Analysis of the function of uptake and release of secretory vesicles in mast cells was performed by quantitative analysis using a normal mercury lamp- or a xenon lamp-based fluorescence ImageXpress Micro microscope (Molecular Devices, Tokyo, Japan). The rapid autofocus and precision sample movement features of the microscope allow large numbers of high-resolution images to be acquired in the shortest possible time. The analysis format can be 96-, 384- or 1536-wells [17]. In the 96-well format using a 20× objective lens, approximately 200 powered fields could be captured at 2.5–5 frames per second for each well using up to a maximum of eight wavelengths. In comparison, image acquisition, processing and analysis of each well would take a long time using a common imaging system.

In the present study, a filter cube with an excitation 375 nm/emission 460 nm pair was used for FFN206-labed vesicle detection. Image acquirement was performed using MetaXpress software (Molecular Devices, Tokyo, Japan) at 37°C in a 5% CO_2_ imaging chamber. At each time point, 9–16 powered fields were captured for each well with 100–400 ms exposure time at a magnification of ×200. All images were 16-bit. Vesicles were identified using a transfluore module of MetaXpress software. The region of interest (ROI) for vesical detection was defined as a diameter range of 2-pixel (0.5 μm) to 10-pixel (2.5 μm) and background subtracted fluorescent intensity ranging from 150-gray scale to 300-gray scale. The total area and integrated intensity were automatically determined at a speed of approximately 1 min per frame. The release of FFN206 from vesicles was calculated according to the following equation: FFN206 release (%) = (total vesicle area before stimulant − total vesicle area after stimulant)/(total vesicle area before stimulant) × 100 or FFN206 release (%) = (vesicle integrated intensity before stimulant − vesicle integrated intensity after stimulant)/(vesicle integrated intensity before stimulant) × 100.

### Measurement of histamine release

RBL-2H3 cells were stimulated with TG at the indicated doses for 30 min. Histamine released into the medium was measured by HPLC-fluorometry [20]. The net release of histamine from cells was calculated according to the following equation: net release (%) = (responsively released histamine − non-responsively leaked histamine)/(total histamine − non-responsively leaked histamine) × 100. Spontaneous release (%) = leaked histamine/total histamine × 100.

### Measurement of released FFN206 in supernatant

RBL-2H3 cells were loaded using FFN206 (5 μM) for 90 min and washed with PIPES buffer (0.1% BSA, pH 7.2). Then, cells were incubated in PIPES buffer at 37°C for the indicated times (5–120 min). After incubation of the cells, the supernatants were transferred to a 96-well imaging plate (Corning, Tokyo, Japan). The concentration of FFN206 in the supernatants was measured using a FlexStation (Molecular Devices, Tokyo, Japan). Fluorescence intensity was measured by detection at an excitation wavelength of 375 nm and emission wavelength of 460 nm.

### Statistical analysis

All experiments were designed in a completely randomized multifactorial format. Results are expressed as the mean ± S.E.M. Pairwise comparisons were performed using a two-tailed Student’s *t*-test to compare TG-dependent histamine release. Two-factor factorial ANOVA followed by the Scheffe F-test were used to compare the time-response curves of increased incubation time to account for unequal variance. *P* values < 0.05 were considered significant.

## Results

### Visualization of FFN206-labeled secretory vesicles in mast cells

**Fig 1:**
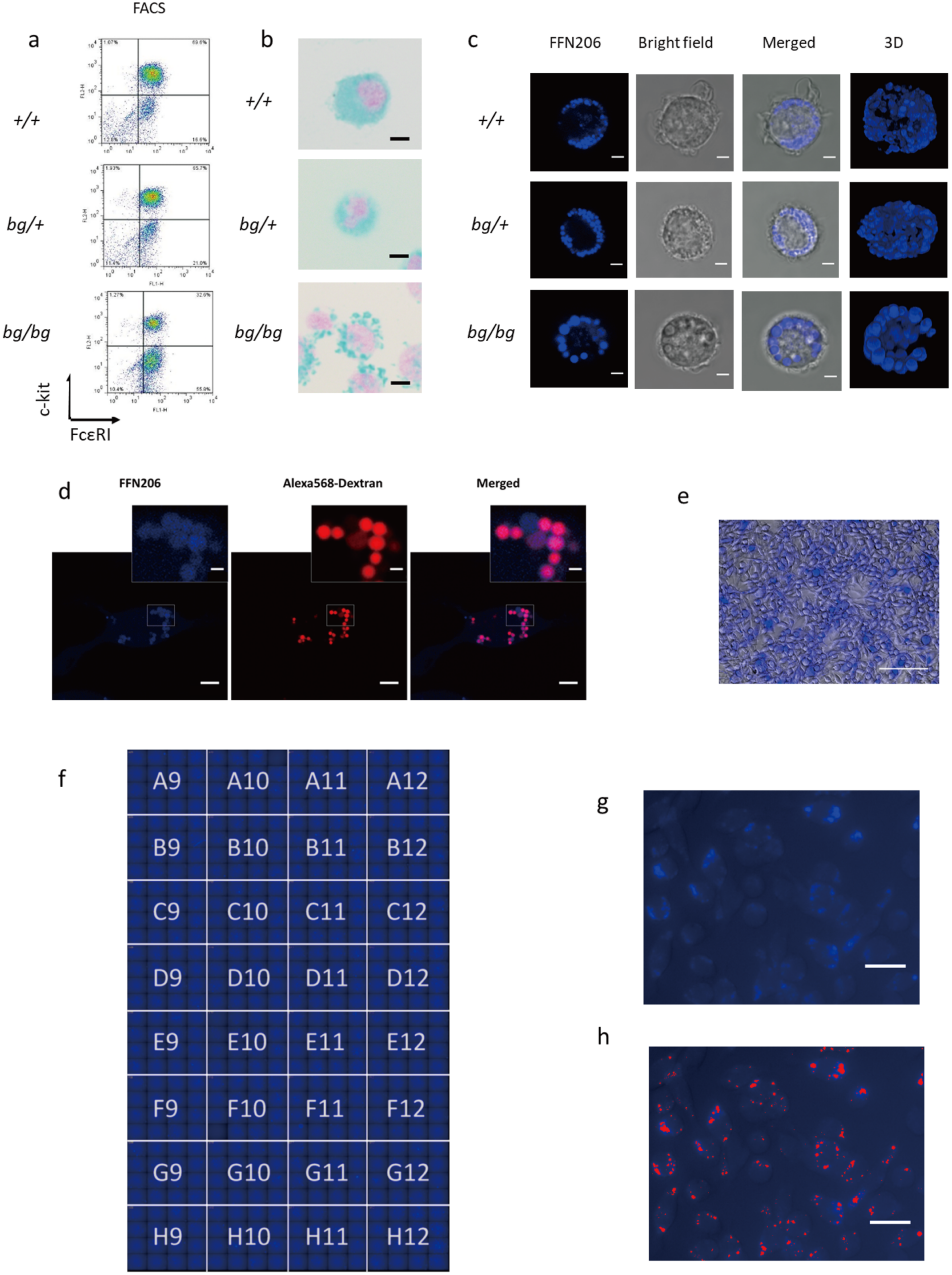
Localization of FFN206-labeled vesicles in mast cells. Primary differentiated bone marrow-derived mast cells (BMMC) derived from *+/+*, *bg/+*, and *bg/bg* mice were used for the detection of secretory vesicles. (a) The expressions of FcεRI receptor and c-kit receptor in BMMC after 4 weeks of culture were analyzed using a BD FACSCalibur flow cytometer. (b) BMMC were stained with Alcian blue after 4 weeks of culture and observed under a light microscope (×400). Scale bar: 5 μm. (c) Visualization of secretory vesicles using FFN206 in BMMC. The cells were observed by confocal microscopy (×1000) at room temperature. Scale bar: 5 μm. (d) Colocalization of FFN206 and Alexa568-dextran in the secretory vesicles of RBL-2H3 cells. The cells were loaded using FFN206 and Alexa568-dextran and were observed by confocal microscopy (×1000) at room temperature. The small images were cropped from original images (gray square) and are present on the upper right of each original image. Scale bar for original image: 10 μm; scale bar for cropped image: 2 μm. (e) Representative image of FFN206-labeled RBL-2H3 cells captured by an ImageXpress Micro microscope at 37°C in a CO_2_ incubator (×200). Scale bar: 100 μm. (f) Imaging map of vesicle analysis in RBL-2H3 cells. Sixteen powered fields were captured for each well. The imaging map was acquired from 32 wells (A9: H12). (g) Representative captured image and (h) segmented vesicles from the background in FFN206-labeled RBL-2H3 cells. Red area: segmented positive area. Scale bar: 20 μm.

Secretory vesicles with different sizes were observed in primary differentiated mast cells derived from *+/+*, *bg/+*, and *bg/bg* mice. *bg/bg* mice are used as a Chediak-Higashi syndrome model and are characterized by enlarged vesicles in mast cells, whereas *bg/+* mice have medium-size vesicles [21–23]. After *ex vivo* differentiation, markers typical of functional mast cells, c-kit and FcεRI, were observed in BMMC (Fig 1a). Vesicles were indistinctly observed in fixed mast cells using traditional Alcian blue staining (Fig 1b). However, using FFN206 labeling, the structural features of different sizes of vesicles were clearly visualized in living mast cells (Fig 1c).

Vesicles were also visualized in RBL-2H3 cells, a cultured mast cell line, and these were used for the development of a high-throughput screening system. The colocalization of FFN206 and Alexa568-dextran were confirmed using confocal microscopy (Fig 1d). In these images, almost all intracellular secretory vesicles were labeled by FFN206 and Alexa568-dextran. For exocytotic screening, the cells were seeded on a 96-well plate. A representative captured image is shown in Fig 1e. Sixteen powered fields were captured for each well. An imaging map acquired from 32 wells is shown in Fig 1f. In a representative captured image (Fig 1g), the vesicles can be easily segregated from the background (Fig 1h).

### Avoiding leakage of FFN206 from vesicles

**Fig 2:**
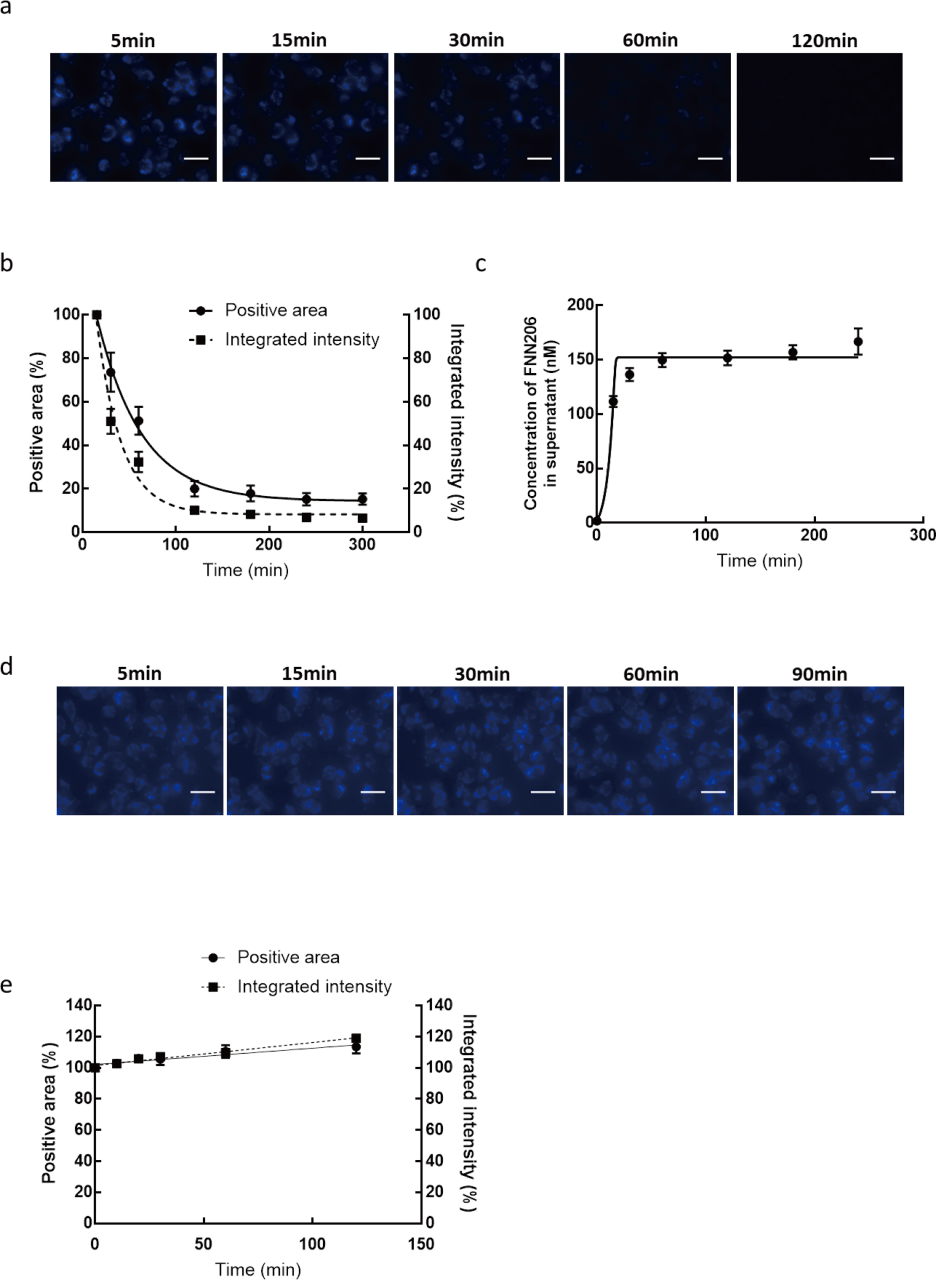
Overcoming leakage of FFN206 from secretory vesicles. RBL-2H3 cells were loaded with FFN206 and observed under an ImageXpress Micro microscope. (a) Representative time serial images of FFN206-loaded RBL-2H3 cells captured with a normal mercury lamp-based ImageXpress Micro microscope (×400) at 15, 30, 60 and 120 min after washing with PIPES buffer. Scale bar: 20 μm. (b) Total positive area (left axis) and integrated intensity (right axis) were quantified based on the captured images. Results are expressed as the mean ± S.E.M. (c) Concentrations of FFN206 in culture supernatants of FFN206-loaded mast cells. After loading, FFN206-loaded cells were washed and cultured in PIPES buffer. Results are expressed as the mean ± S.E.M. (d) Representative time serial images of FFN206-loaded RBL-2H3 cells captured with a xenon lamp-based ImageXpress Micro microscope (×400) at 15, 30, 60 and 120 min. Extracellular FFN206 was maintained after loading without washing out. The cells were observed using a xenon lamp instead of a normal mercury lamp. Scale bar: 20 μm. (e) Total positive area (left axis) and integrated intensity (right axis) were quantified based on the captured images using an optimized protocol. Results are expressed as the mean ± S.E.M.

During the development of a vesicle trafficking screening system based on FFN206 labeling, a problem emerged. We found that the fluorescence intensity of labeled vesicles rapidly declined after washing with PIPES buffer (Fig 2a). Both the positive area and integrated intensity of observed vesicles quickly decreased in a time-dependent manner (Fig 2b). According to the Asymmetrical (five parameter) equation described (Model: LogXb = LogEC50 + (1/HillSlope)*Log((2^(1/S))-1); Numerator = Top − Bottom; Denominator = (1+10^((LogXb-X)*HillSlope))^S; Y = Bottom + (Numerator/Denominator)), the logistic effective time (*logET*_*50*_) was 28.8 ± 2.52 min, the *HillSlope* was −0.2237, *S* was 0.041, *Top* was 142.5 and the *Bottom* was 14.34 for the positive area. For the integrated intensity the *logET*_*50*_ was 22.73 ± 1.06 min, the *HillSlope* was −0.4, *S* was 0.04, *Top* was 139.3 and the *Bottom* was 8.26 f. Moreover, the concentration of FFN206 in the supernatant (PIPES) reached a plateau at 60 min with a *logET50* of 12.36 ± 1.27 min (Fig 2c). These results suggest that leakage or fluorescence bleaching can occur during FFN206 labeling.

To overcome this problem, we attempted to maintain the extracellular FFN 206 after loading without washing out. Using a xenon lamp instead of a normal mercury lamp, vesicles could still be distinguished and segmented from captured images with high background fluorescence (Fig 2d). During the whole period of observation, a stable fluorescence level was maintained in secretory vesicles (Fig 2e). Therefore, this optimized protocol was used for further dynamic screening experiments.

### Detection of functional vesicle uptake via VMAT2

**Fig 3:**
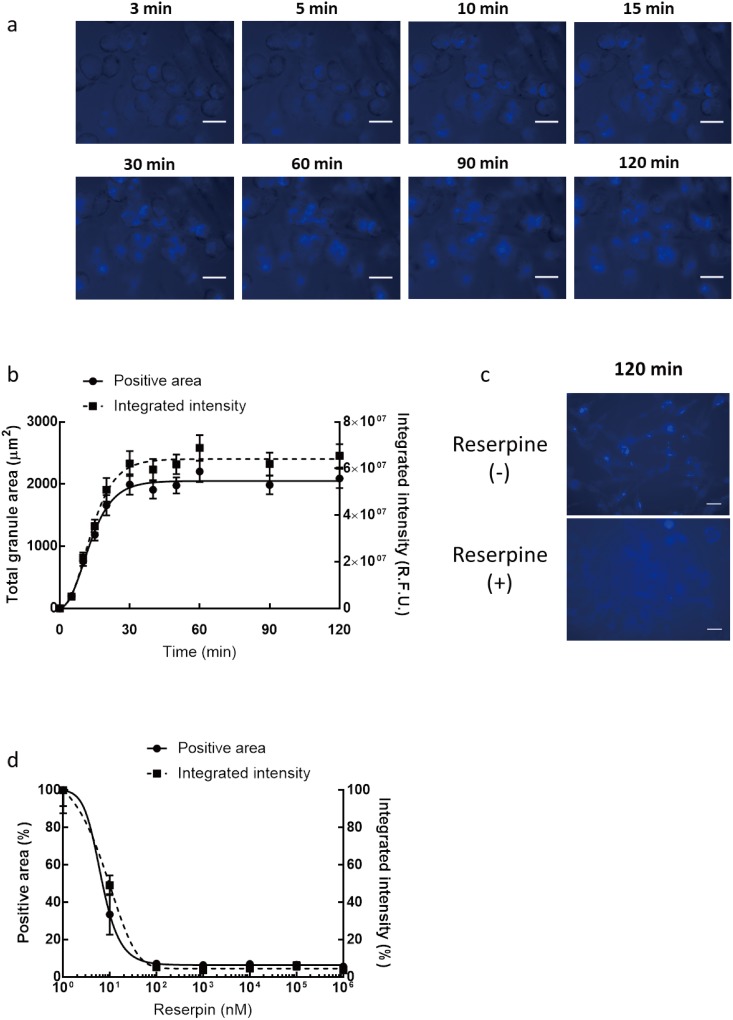
Detection of function of vesicle uptake in secretory vesicles. (a) FFN206 was added to RBL-2H3 cells and the uptake process was observed under an ImageXpress Micro microscope (×400). Scale bar: 20 μm. (b) Total positive area (left axis) and integrated intensity (right axis) to loading time were quantified based on the captured images. Results are expressed as the mean ± S.E.M. (c) Representative uptake images of FFN206 in non-treated and reserpine-treated cells 90 min after FFN206 application (×400). Scale bar: 20 μm. (d) Blockade of functional vesicle uptake by the administration of reserpine in mast cells. The cells were pre-treated with reserpine at the indicated doses and FFN206 loading was performed. The maximal positive area (left axis) and integrated intensity (right axis) at different doses of reserpine were quantified based on the captured images. Results are expressed as the mean ± S.E.M.

We monitored the uptake function of vesicles using an established screening system. Time-dependent internalization of FFN206 into vesicles was analyzed (Fig 3a). Increases of positive area and integrated intensity were observed with a *logET50* of 12.66 ± 0.92 min for the positive area and 13.23 ± 0.97 min for the integrated intensity (Fig 3b). After pretreatment with reserpine, an irreversible non-specific VMAT1/2 inhibitor, reserpine dependence of vesicle uptake was observed (Fig 3c). The effective dose (*EC50*) was calculated with *logEC50* of 0.81 ± 0.13 nM for the positive area and *logEC50* of 0.88 ± 0.67 nM for the integrated intensity (Fig 3d). This result suggests that the function of vesicle uptake in mast cells is significantly blocked by reserpine in a dose-dependent manner.

### Dynamic observation of exocytotic trafficking in mast cells

**Fig 4:**
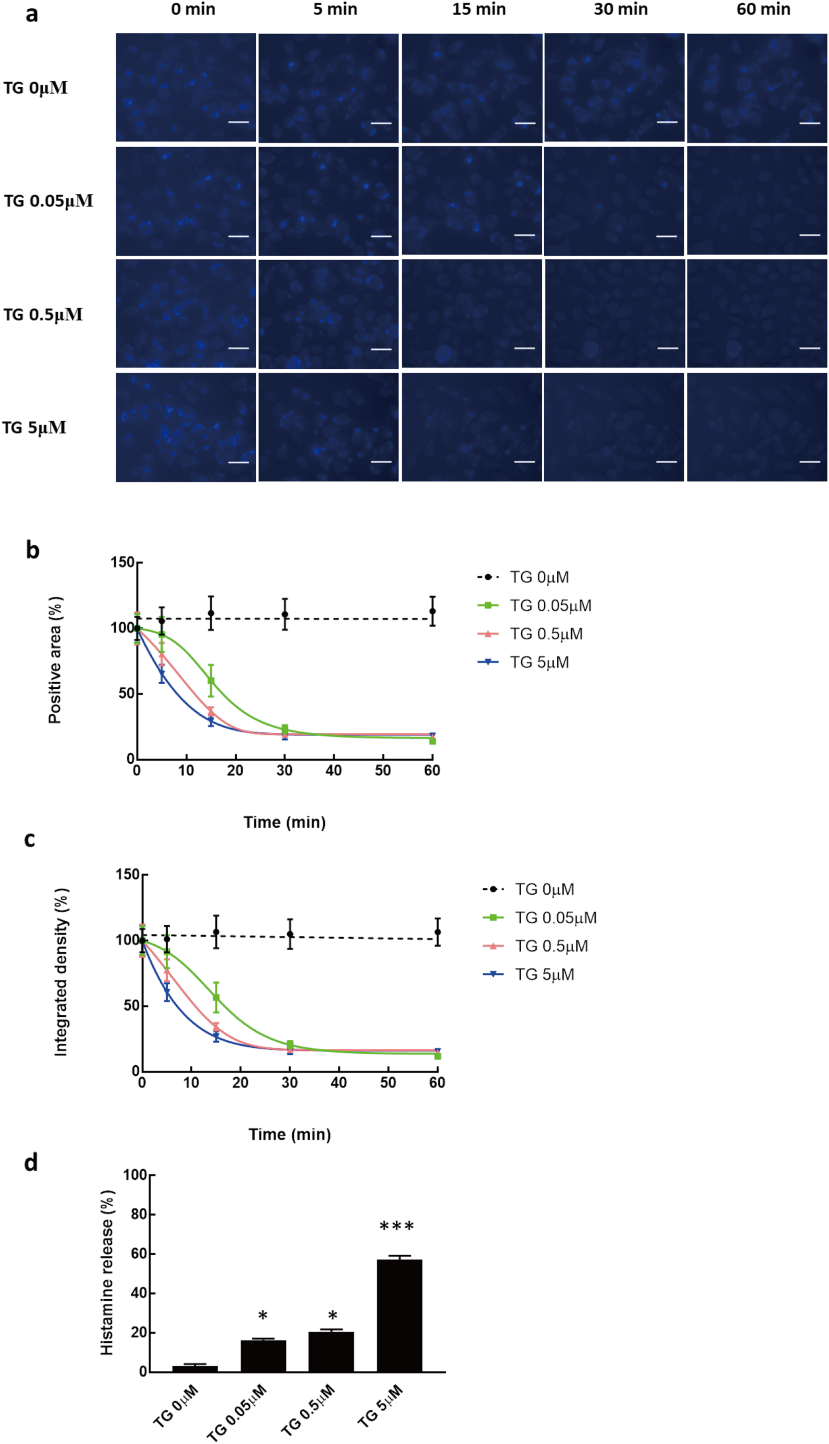
Observation of exocytotic trafficking in mast cells. FFN206-loaded RBL-2H3 cells were stimulated with different concentrations of thapsigargin (0.05, 0.5 and 5 μM). Exocytotic processes were monitored. (a) Representative images of TG-activated mast cells captured by an ImageXpress Micro microscope (×400). Scale bar: 20 μm. (b) Rate of exocytosis quantified using the total positive area based on the captured images. Results are expressed as the mean ± S.E.M. (c) The rate of exocytosis was quantified using the integrated intensity based on the captured images. Results are expressed as the mean ± S.E.M. (d) TG-dependent histamine release was measured 30 min after stimulation. Results are expressed as the mean ± S.E.M. *p < 0.05; ***p < 0.001.

The rate of exocytotic trafficking was quantified in mast cells. Different doses of TG were used as stimulators to trigger the exocytotic process. Typical captured images are shown in Fig 4a. The rate curves of both the positive area and integrated intensity in TG-treated mast cells were shifted to the left dose-dependently (Fig 4b & 4c). According to the Asymmetrical (five parameter) equation described, exocytosis curves were obtained with a *logET50* of 13.23 min in 0.05 μM TG-treated cells, 5.32 min in 0.5 μM TG-treated cells and 0.97 min in 5 μM TG-treated cells, showing that TG activity increased the rate of exocytotic trafficking dose-dependently. Similar results were also observed for integrated intensity quantification with a *logET50* of 14.9 min in 0.05 μM TG-treated cells, 4.13 min in 0.5 μM TG-treated cells and 0.93 min in 5 μM TG-treated cells. Histamine release, which is commonly used to evaluate exocytotic function in mast cells, was measured 30 min after TG treatment at the indicated doses (Fig 4d). Histamine release was 16.33 ± 0.80% in 0.05 μM TG-treated cells, 20.52 ± 1.33% in 0.5 μM TG-treated cells and 57.10 ± 2.10% in 5 μM TG-treated cells. These results confirm the outcome of our imaging screening that TG triggers exocytosis dose-dependently in mast cells.

## Discussion

In the present study, we established a real-time high-throughput screening method to monitor exocytotic trafficking in mast cells. Using this method, the function of vesicle uptake and stimuli-dependent exocytosis were dynamically analyzed by real-time fluorescence quantitation.

The images of primary differentiated BMMC and cultured RBL-2H3 cells suggested that labeling using FFN206 is a useful technique for the morphological and functional studies of living mast cells. The effective labeling with FFN206 fluorescence, which is stable in acidic conditions, was independent of the type and size of secretory vesicles, as we observed in *+/+*, *bg/+* and *bg/bg* mice-derived mast cells [17].

Of note, intra-vesicle florescence intensity was not stably maintained after FFN206 loading and a significant leakage of FFN206 was detected after washing with PIPES buffer. This explains why a previous report recommended that FFN206-labeled vesicles should be observed promptly [17]. The phenomenon whereby FFN206 was transported from vesicles to the extracellular space in loaded cells not only occurred in mast cells but also in central neurons and other cells. ATP binding cassette (ABC) proteins, which maintain physiological roles by transporting hydrophobic compounds [24], may be involved in the clearance of FFN206 from vesicles. Multidrug resistance protein (MDR1), a member of the ABC protein family, transports a large number of structurally unrelated compounds and is therefore involved in their pharmacokinetics [25]. It was reported that the *MDR1* gene is expressed in two canine mast cell tumor cell lines, CoMC and LuMC [26]. Blockade of ABC proteins may help inhibit the leakage of FFN206 from intracellular vesicles. In the present system, we overcame the problem of leakage by removing the gradient of the fluorescent indicator. By maintaining extracellular FFN206, the leakage of fluorescence from vesicles is avoided. Moreover, a xenon lamp, which was used to detect FFN206-aggregated vesicles in the present study, overcame the obstacle of high background fluorescence.

VMAT2 is expressed on the surface of secretory vesicles in mast cells as well as central neurons [11]. Histamine and other mediators were taken up time-dependently and reached a maximal level within 30–40 min in VMAT2-expressing cells [27]. Using our dynamic screening system, the time-dependent uptake of FFN206 was monitored and the maximal level was observed at 30 min after loading. The uptake function was blocked by the administration of reserpine, which also blocked the uptake of neuronal transmitters in monoaminergic neurons [17]. These results suggest that FFN206 may share a similar uptake mechanism with histamine and neurotransmitters. Our screening system also allows the real-time monitoring of exocytotic trafficking, which is difficult using traditional bioassay and cell staining methods. The velocities of TG-triggered exocytotic processes can be quantified by the decrease of positive area and integrated intensity.

Interestingly, a discrepancy occurred in the interpretation of results obtained from the imaging technique and from the bioassay. After 30 min, the positive area or the integrated density for mast cells treated with different concentrations of TG were reduced to a similar level, in contrast to the amounts of histamine released under similar conditions. The amount of histamine released when the concentration of TG was 0.05 or 0.5 μM was approximately 20%, which increased to almost 60% at 5 μM TG. The following possible reasons may explain this discrepancy. First, FNN206 may not label all intracellular secretory vesicles and histamine releasing potentials are still maintained in those unlabeled vesicles. In the present study, the loading period for FFN206 was 90 min. In this period, whether all FFN206 was sufficiently taken up by the secretory vesicles is unclear. This should be determined in the future to dissect the relationship between the loading protocol and exocytosis. Second, the expression level of VMAT2, a transporter for FFN206 loading into vesicles, might vary in individual vesicles at different stages of formation. The expression level of VMAT2 may be associated with the packaging and storage activity of the vesicles and intracellular monoamine concentration [28]. This differential expression of VMAT2 related to histamine storage stage and FFN206 loading stage might explain the different results obtained by imaging assay and bioassay. Our future study will investigate the relationship between vesicle formation status and amine uptake using immune election microscopic techniques. Furthermore, the release of histamine newly taken up from the extracellular compartment, but not intracellular synthesized and pre-stored histamine in vesicles, may mimic the situation with FFN206. Taken together, the imaging dynamic screening system of FFN206 labeled vesicles shows a snapshot but not the full picture of exocytosis. Two appropriately combined techniques might enhance the accuracy of our exocytotic observations.

In conclusion, the current study established a high-throughput imaging screening system for the monitoring of functional vesicles in mast cells. This screening system was used to monitor the uptake and exocytotic trafficking of vesicles. When using this method for the screening of mast cell activation, the prevention of indicator leakage is critical. Along with traditional functional bioassay systems, our new imaging tool might benefit drug innovation targeting intracellular vesicle trafficking in mast cells.
